# Supplementary material for: Remote monitoring, personnel extinguishment or helicopter extinguishment? How to control forest fires more effectively
Source: PLoS One. 2023 Aug 10;18(8):e0289727. doi: 10.1371/journal.pone.0289727 (PMC10414624; doi:10.1371/journal.pone.0289727)
Supplement: S1 File — (DOCX) [file pone.0289727.s001.docx]

Appendix 1

Take the derivatives of *M_R_*_1_ and *T_R_*_1_ respectively with respect to (13), and take the derivatives of *E_R_*_2_ with respect to (14), and set them equal to zero, we can get:

$M_{R1}^{*}\left(t\right)=\frac{b_{M1}\ln\left(1+w_{S}\right)+a_{1}\frac{\partial V_{R1}}{\partial x_{R1}}}{c_{M1}}$ (44)

$T_{R1}^{\text{*}}\left(t\right)=\frac{c_{T1}^{2}}{16\left(b_{T1}+a_{1}\frac{\partial V_{R1}}{\partial x_{R1}}\right)^{2}}$ (45)

$E_{R2}^{*}\left(t\right)=\frac{v_{w}^{2}d_{R2}^{2}}{\ln^{2}\left(e+I_{R2}\right)\left[2b_{E}+2\frac{\partial V_{R2}}{\partial x_{R2}}a_{2}\ln\left(1+I_{R2}\right)\right]^{2}}$ (46)

Substituting (44) and (45) into (13) and substituting (46) into (14), we can get:

$\begin{matrix} \rho V_{R1}=b_{M1}\frac{b_{M1}\ln\left(1+w_{S}\right)+a_{1}\frac{\partial V_{R1}}{\partial x_{R1}}}{c_{M1}}\ln\left(1+w_{S}\right)-\frac{c_{M1}}{2}\left[\frac{b_{M1}\ln\left(1+w_{S}\right)+a_{1}\frac{\partial V_{R1}}{\partial x_{R1}}}{c_{M1}}\right]^{2} \\ +b_{T1}\frac{c_{T1}^{2}}{16\left(b_{T1}+a_{1}\frac{\partial V_{R1}}{\partial x_{R1}}\right)^{2}}-\frac{c_{T1}}{2}\frac{c_{T1}}{4\left(b_{T1}+a_{1}\frac{\partial V_{R1}}{\partial x_{R1}}\right)}+lx_{R1}\left(t\right) \\ +\frac{\partial V_{R1}}{\partial x_{R1}}a_{1}\left(\frac{b_{M1}\ln\left(1+w_{S}\right)+a_{1}\frac{\partial V_{R1}}{\partial x_{R1}}}{c_{M1}}+\frac{c_{T1}^{2}}{16\left(b_{T1}+a_{1}\frac{\partial V_{R1}}{\partial x_{R1}}\right)^{2}}\right)-\frac{\partial V_{R1}}{\partial x_{R1}}\delta x_{R1}\left(t\right) \end{matrix}$ (47)

$\begin{matrix} \rho V_{R2}=-\frac{v_{w}d_{R2}}{\ln\left(e+I_{R2}\right)}\frac{v_{w}d_{R2}}{\ln\left(e+I_{R2}\right)\left[2b_{E}+2\frac{\partial V_{R2}}{\partial x_{R2}}a_{2}\ln\left(1+I_{R2}\right)\right]} \\ +\frac{b_{E}v_{w}^{2}d_{R2}^{2}}{\ln^{2}\left(e+I_{R2}\right)\left[2b_{E}+2\frac{\partial V_{R2}}{\partial x_{R2}}a_{2}\ln\left(1+I_{R2}\right)\right]^{2}}-\frac{\partial V_{R2}}{\partial x_{R2}}\delta x_{R2}\left(t\right)+lx_{R2}\left(t\right) \\ +\frac{\partial V_{R2}}{\partial x_{R2}}a_{2}\frac{v_{w}^{2}d_{R2}^{2}}{\ln^{2}\left(e+I_{R2}\right)\left[2b_{E}+2\frac{\partial V_{R2}}{\partial x_{R2}}a_{2}\ln\left(1+I_{R2}\right)\right]^{2}}\ln\left(1+I_{R2}\right) \end{matrix}$ (48)

Let $V_{R1}^{*}=k_{1}x_{R1}+k_{2}$,$V_{R2}^{\text{*}}=k_{3}x_{R2}+k_{4}$, wherein, *k*_1_, *k*_2_, *k*_3_ and *k*_4_ are all constants. The parameters of the optimal social welfare function can be obtained by calculation as follows:

$\left\{\begin{matrix} k_{1}=\frac{l}{\rho+\delta} \\ k_{2}=\frac{1}{\rho}b_{M1}\frac{b_{M1}\ln\left(1+w_{S}\right)+a_{1}\frac{l}{\rho+\delta}}{c_{M1}}\ln\left(1+w_{S}\right)-\frac{1}{\rho}\frac{c_{M1}}{2}\left[\frac{b_{M1}\ln\left(1+w_{S}\right)+a_{1}\frac{l}{\rho+\delta}}{c_{M1}}\right]^{2} \\ +\frac{1}{\rho}b_{T1}\frac{c_{T1}^{2}}{16\left(b_{T1}+a_{1}\frac{l}{\rho+\delta}\right)^{2}}-\frac{c_{T1}}{2}\frac{c_{T1}}{4\left(b_{T1}+a_{1}\frac{l}{\rho+\delta}\right)} \\ +\frac{1}{\rho}\frac{l}{\rho+\delta}a_{1}\left(\frac{b_{M1}\ln\left(1+w_{S}\right)+a_{1}\frac{l}{\rho+\delta}}{c_{M1}}+\frac{c_{T1}^{2}}{16\left(b_{T1}+a_{1}\frac{l}{\rho+\delta}\right)^{2}}\right) \end{matrix}\right.$ (49)

$\left\{\begin{matrix} k_{3}=\frac{l}{\rho+\delta} \\ k_{4}=-\frac{1}{\rho}\frac{v_{w}d_{R2}}{\ln\left(e+I_{R2}\right)}\frac{v_{w}d_{R2}}{\ln\left(e+I_{R2}\right)\left[2b_{E}+2\frac{l}{\rho+\delta}a_{2}\ln\left(1+I_{R2}\right)\right]} \\ +\frac{1}{\rho}\frac{b_{E}v_{w}^{2}d_{R2}^{2}}{\ln^{2}\left(e+I_{R2}\right)\left[2b_{E}+2\frac{l}{\rho+\delta}a_{2}\ln\left(1+I_{R2}\right)\right]^{2}} \\ +\frac{1}{\rho}\frac{l}{\rho+\delta}a_{2}\frac{v_{w}^{2}d_{R2}^{2}}{\ln^{2}\left(e+I_{R2}\right)\left[2b_{E}+2\frac{l}{\rho+\delta}a_{2}\ln\left(1+I_{R2}\right)\right]^{2}}\ln\left(1+I_{R2}\right) \end{matrix}\right.$ (50)

Therefore, it can be concluded that:

$\begin{matrix} V_{R1}^{*}=\frac{l}{\rho+\delta}x_{R1}+\frac{1}{\rho}b_{M1}\frac{b_{M1}\ln\left(1+w_{S}\right)+a_{1}\frac{l}{\rho+\delta}}{c_{M1}}\ln\left(1+w_{S}\right)-\frac{1}{\rho}\frac{c_{M1}}{2}\left[\frac{b_{M1}\ln\left(1+w_{S}\right)+a_{1}\frac{l}{\rho+\delta}}{c_{M1}}\right]^{2} \\ +\frac{1}{\rho}b_{T1}\frac{c_{T1}^{2}}{16\left(b_{T1}+a_{1}\frac{l}{\rho+\delta}\right)^{2}}-\frac{c_{T1}}{2}\frac{c_{T1}}{4\left(b_{T1}+a_{1}\frac{l}{\rho+\delta}\right)} \\ +\frac{1}{\rho}\frac{l}{\rho+\delta}a_{1}\left(\frac{b_{M1}\ln\left(1+w_{S}\right)+a_{1}\frac{l}{\rho+\delta}}{c_{M1}}+\frac{c_{T1}^{2}}{16\left(b_{T1}+a_{1}\frac{l}{\rho+\delta}\right)^{2}}\right) \end{matrix}$(51)

$\begin{matrix} V_{R2}^{\text{*}}=\frac{l}{\rho+\delta}x_{R2}-\frac{1}{\rho}\frac{v_{w}d_{R2}}{\ln\left(e+I_{R2}\right)}\frac{v_{w}d_{R2}}{\ln\left(e+I_{R2}\right)\left[2b_{E}+2\frac{l}{\rho+\delta}a_{2}\ln\left(1+I_{R2}\right)\right]} \\ +\frac{1}{\rho}\frac{b_{E}v_{w}^{2}d_{R2}^{2}}{\ln^{2}\left(e+I_{R2}\right)\left[2b_{E}+2\frac{l}{\rho+\delta}a_{2}\ln\left(1+I_{R2}\right)\right]^{2}} \\ +\frac{1}{\rho}\frac{l}{\rho+\delta}a_{2}\frac{v_{w}^{2}d_{R2}^{2}}{\ln^{2}\left(e+I_{R2}\right)\left[2b_{E}+2\frac{l}{\rho+\delta}a_{2}\ln\left(1+I_{R2}\right)\right]^{2}}\ln\left(1+I_{R2}\right) \end{matrix}$ (52)

In this case,

$M_{R1}^{*}\left(t\right)=\frac{b_{M1}\ln\left(1+w_{S}\right)+a_{1}\frac{l}{\rho+\delta}}{c_{M1}}$ (53)

$T_{R1}^{\text{*}}\left(t\right)=\frac{c_{T1}^{2}}{16\left(b_{T1}+a_{1}\frac{l}{\rho+\delta}\right)^{2}}$ (54)

$E_{R2}^{*}\left(t\right)=\frac{v_{w}^{2}d_{R2}^{2}}{\ln^{2}\left(e+I_{R2}\right)\left[2b_{E}+2\frac{l}{\rho+\delta}a_{2}\ln\left(1+I_{R2}\right)\right]^{2}}$ (55)

Appendix 2

Take the derivatives of *M_P_*_1_ with respect to (15), and take the derivatives of *E_P_*_2_ with respect to (16), and set them equal to zero, we can get:

$M_{P1}^{\text{*}}\left(t\right)=\frac{b_{M1}-v_{w}q_{P1}+\frac{\partial V_{P1}}{\partial x_{P1}}a_{1}}{c_{M1}}$ (56)

$E_{P2}^{*}\left(t\right)=\frac{v_{w}^{2}d_{P2}^{2}}{4\left(b_{E}+\frac{\partial V_{P2}}{\partial x_{P2}}a_{2}\right)^{2}}$ (57)

Substituting (56) into (15) and substituting (57) into (16), we can get:

$\begin{matrix} \rho V_{P1}=b_{M1}\frac{b_{M1}-v_{w}q_{P1}+\frac{\partial V_{P1}}{\partial x_{P1}}a_{1}}{c_{M1}}-v_{w}q_{P1}\frac{b_{M1}-v_{w}q_{P1}+\frac{\partial V_{P1}}{\partial x_{P1}}a_{1}}{c_{M1}}-\frac{c_{M1}}{2}\left(\frac{b_{M1}-v_{w}q_{P1}+\frac{\partial V_{P1}}{\partial x_{P1}}a_{1}}{c_{M1}}\right)^{2} \\ +\frac{\partial V_{P1}}{\partial x_{P1}}a_{1}\frac{b_{M1}-v_{w}q_{P1}+\frac{\partial V_{P1}}{\partial x_{P1}}a_{1}}{c_{M1}}-\delta x_{P1}\left(t\right)\frac{\partial V_{P1}}{\partial x_{P1}}+lx_{P1}\left(t\right) \end{matrix}$ (58)

$\begin{matrix} \rho V_{P2}=-v_{w}d_{P2}\frac{v_{w}d_{P2}}{2\left(b_{E}+\frac{\partial V_{P2}}{\partial x_{P2}}a_{2}\right)}+b_{E}\frac{v_{w}^{2}d_{P2}^{2}}{4\left(b_{E}+\frac{\partial V_{P2}}{\partial x_{P2}}a_{2}\right)^{2}}+lx_{P2}\left(t\right) \\ +\frac{\partial V_{P2}}{\partial x_{P2}}a_{2}\frac{v_{w}^{2}d_{P2}^{2}}{4\left(b_{E}+\frac{\partial V_{P2}}{\partial x_{P2}}a_{2}\right)^{2}}-\delta x_{P2}\left(t\right)\frac{\partial V_{P2}}{\partial x_{P2}} \end{matrix}$ (59)

Let $V_{P1}^{*}=k_{5}x_{P1}+k_{6}$,$V_{P2}^{\text{*}}=k_{7}x_{P2}+k_{8}$, wherein, *k*_5_, *k*_6_, *k*_7_ and *k*_8_ are all constants. The parameters of the optimal social welfare function can be obtained by calculation as follows:

$\left\{\begin{matrix} k_{5}=\frac{l}{\rho+\delta} \\ k_{6}=\frac{1}{\rho}b_{M1}\frac{b_{M1}-v_{w}q_{P1}+\frac{l}{\rho+\delta}a_{1}}{c_{M1}}-\frac{1}{\rho}v_{w}q_{P1}\frac{b_{M1}-v_{w}q_{P1}+\frac{l}{\rho+\delta}a_{1}}{c_{M1}} \\ -\frac{1}{\rho}\frac{c_{M1}}{2}\left(\frac{b_{M1}-v_{w}q_{P1}+\frac{l}{\rho+\delta}a_{1}}{c_{M1}}\right)^{2}+\frac{1}{\rho}\frac{l}{\rho+\delta}a_{1}\frac{b_{M1}-v_{w}q_{P1}+\frac{l}{\rho+\delta}a_{1}}{c_{M1}} \end{matrix}\right.$ (60)

$\left\{\begin{matrix} k_{7}=\frac{l}{\rho+\delta} \\ k_{8}=-\frac{1}{\rho}v_{w}d_{P2}\frac{v_{w}d_{P2}}{2\left(b_{E}+\frac{l}{\rho+\delta}a_{2}\right)}+b_{E}\frac{1}{\rho}\frac{v_{w}^{2}d_{P2}^{2}}{4\left(b_{E}+\frac{l}{\rho+\delta}a_{2}\right)^{2}} \\ +\frac{1}{\rho}\frac{l}{\rho+\delta}a_{2}\frac{v_{w}^{2}d_{P2}^{2}}{4\left(b_{E}+\frac{l}{\rho+\delta}a_{2}\right)^{2}} \end{matrix}\right.$ (61)

$\begin{matrix} V_{P1}^{*}=\frac{l}{\rho+\delta}x_{P1}+\frac{1}{\rho}b_{M1}\frac{b_{M1}-v_{w}q_{P1}+\frac{l}{\rho+\delta}a_{1}}{c_{M1}}-\frac{1}{\rho}v_{w}q_{P1}\frac{b_{M1}-v_{w}q_{P1}+\frac{l}{\rho+\delta}a_{1}}{c_{M1}} \\ -\frac{1}{\rho}\frac{c_{M1}}{2}\left(\frac{b_{M1}-v_{w}q_{P1}+\frac{l}{\rho+\delta}a_{1}}{c_{M1}}\right)^{2}+\frac{1}{\rho}\frac{l}{\rho+\delta}a_{1}\frac{b_{M1}-v_{w}q_{P1}+\frac{l}{\rho+\delta}a_{1}}{c_{M1}} \end{matrix}$ (62)

$\begin{matrix} V_{P2}^{\text{*}}=\frac{l}{\rho+\delta}x_{P2}-\frac{1}{\rho}v_{w}d_{P2}\frac{v_{w}d_{P2}}{2\left(b_{E}+\frac{l}{\rho+\delta}a_{2}\right)}+b_{E}\frac{1}{\rho}\frac{v_{w}^{2}d_{P2}^{2}}{4\left(b_{E}+\frac{l}{\rho+\delta}a_{2}\right)^{2}} \\ +\frac{1}{\rho}\frac{l}{\rho+\delta}a_{2}\frac{v_{w}^{2}d_{P2}^{2}}{4\left(b_{E}+\frac{l}{\rho+\delta}a_{2}\right)^{2}} \end{matrix}$ (63)

In this case,

$M_{P1}^{\text{*}}\left(t\right)=\frac{b_{M1}-v_{w}q_{P1}+\frac{l}{\rho+\delta}a_{1}}{c_{M1}}$ (64)

$E_{P2}^{*}\left(t\right)=\frac{v_{w}^{2}d_{P2}^{2}}{4\left(b_{E}+\frac{l}{\rho+\delta}a_{2}\right)^{2}}$ (65)

Appendix 3

Take the derivatives of *T_H_*_1_ with respect to (17), and take the derivatives of *E_H_*_2_ with respect to (18), and set them equal to zero, we can get:

$T_{H1}^{\text{*}}\left(t\right)=\frac{b_{T1}+\frac{\partial V_{H1}}{\partial x_{H1}}a_{1}}{2dv_{w}}$ (66)

$E_{H2}^{*}\left(t\right)=\frac{v_{w}^{2}d_{H2}^{2}}{4\left[b_{E}\ln\left(1+P_{H2}\right)+\frac{\partial V_{H2}}{\partial x_{H2}}a_{2}\right]^{2}}$ (67)

Substituting (66) into (17) and substituting (67) into (18), we can get:

$\rho V_{H1}=b_{T1}\frac{b_{T1}+\frac{\partial V_{H1}}{\partial x_{H1}}a_{1}}{2dv_{w}}-dv_{w}\left(\frac{b_{T1}+\frac{\partial V_{H1}}{\partial x_{H1}}a_{1}}{2dv_{w}}\right)^{2}+lx_{H1}\left(t\right)+\frac{\partial V_{H1}}{\partial x_{H1}}a_{1}\frac{b_{T1}+\frac{\partial V_{H1}}{\partial x_{H1}}a_{1}}{2dv_{w}}-\delta x_{H1}\left(t\right)\frac{\partial V_{H1}}{\partial x_{H1}}$ (68)

$\begin{matrix} \rho V_{H2}=-v_{w}d_{H2}\frac{v_{w}d_{H2}}{2\left[b_{E}\ln\left(1+P_{H2}\right)+\frac{\partial V_{H2}}{\partial x_{H2}}a_{2}\right]}+b_{E}\frac{v_{w}^{2}d_{H2}^{2}}{4\left[b_{E}\ln\left(1+P_{H2}\right)+\frac{\partial V_{H2}}{\partial x_{H2}}a_{2}\right]^{2}}\ln\left(\text{e}+p_{H2}\right) \\ +\frac{\partial V_{H2}}{\partial x_{H2}}a_{2}\frac{v_{w}^{2}d_{H2}^{2}}{4\left[b_{E}\ln\left(1+P_{H2}\right)+\frac{\partial V_{H2}}{\partial x_{H2}}a_{2}\right]^{2}}-\delta x_{H2}\left(t\right)\frac{\partial V_{H2}}{\partial x_{H2}}+lx_{H2}\left(t\right) \end{matrix}$ (69)

Let $V_{H1}^{*}=k_{9}x_{H1}+k_{10}$,$V_{H2}^{\text{*}}=k_{11}x_{H2}+k_{12}$, wherein, *k*_9_, *k*_10_, *k*_11_ and *k*_12_ are all constants. The parameters of the optimal social welfare function can be obtained by calculation as follows:

$\left\{\begin{matrix} k_{9}=\frac{l}{\rho+\delta} \\ k_{10}=\frac{1}{\rho}b_{T1}\frac{b_{T1}+\frac{l}{\rho+\delta}a_{1}}{2dv_{w}}-\frac{1}{\rho}dv_{w}\left(\frac{b_{T1}+\frac{l}{\rho+\delta}a_{1}}{2dv_{w}}\right)^{2}+\frac{1}{\rho}\frac{l}{\rho+\delta}a_{1}\frac{b_{T1}+\frac{l}{\rho+\delta}a_{1}}{2dv_{w}} \end{matrix}\right.$ (70)

$\left\{\begin{matrix} k_{11}=\frac{l}{\rho+\delta} \\ k_{12}=-\frac{1}{\rho}v_{w}d_{H2}\frac{v_{w}d_{H2}}{2\left[b_{E}\ln\left(1+P_{H2}\right)+\frac{l}{\rho+\delta}a_{2}\right]}+\frac{1}{\rho}b_{E}\frac{v_{w}^{2}d_{H2}^{2}}{4\left[b_{E}\ln\left(1+P_{H2}\right)+\frac{l}{\rho+\delta}a_{2}\right]^{2}}\ln\left(\text{e}+p_{H2}\right) \\ +\frac{1}{\rho}\frac{l}{\rho+\delta}a_{2}\frac{v_{w}^{2}d_{H2}^{2}}{4\left[b_{E}\ln\left(1+P_{H2}\right)+\frac{l}{\rho+\delta}a_{2}\right]^{2}} \end{matrix}\right.$(71)

$V_{H1}^{*}=\frac{l}{\rho+\delta}x_{H1}+\frac{1}{\rho}b_{T1}\frac{b_{T1}+\frac{l}{\rho+\delta}a_{1}}{2dv_{w}}-\frac{1}{\rho}dv_{w}\left(\frac{b_{T1}+\frac{l}{\rho+\delta}a_{1}}{2dv_{w}}\right)^{2}+\frac{1}{\rho}\frac{l}{\rho+\delta}a_{1}\frac{b_{T1}+\frac{l}{\rho+\delta}a_{1}}{2dv_{w}}$ (72)

$\begin{matrix} V_{H2}^{\text{*}}=\frac{l}{\rho+\delta}x_{H2}-\frac{1}{\rho}v_{w}d_{H2}\frac{v_{w}d_{H2}}{2\left[b_{E}\ln\left(1+P_{H2}\right)+\frac{l}{\rho+\delta}a_{2}\right]}+\frac{1}{\rho}b_{E}\frac{v_{w}^{2}d_{H2}^{2}}{4\left[b_{E}\ln\left(1+P_{H2}\right)+\frac{l}{\rho+\delta}a_{2}\right]^{2}} \\ \ln\left(\text{e}+p_{H2}\right)+\frac{1}{\rho}\frac{l}{\rho+\delta}a_{2}\frac{v_{w}^{2}d_{H2}^{2}}{4\left[b_{E}\ln\left(1+P_{H2}\right)+\frac{l}{\rho+\delta}a_{2}\right]^{2}} \end{matrix}$ (73)
